# First Brazilian Symposium of Frontotemporal Lobar Degeneration

**Published:** 2007

## ABSTRACT-1

**The frontal lobes and the limbic system**

Martin Metzger

Department of Physiology & Biophysics, Institute of Biomedical Sciences, USP, São Paulo, Brazil.

The frontal lobes are constituted by the prefrontal cortex (PFC), the pre-motor areas and the primary motor cortex. The prefrontal cortex is situated at the apex of the perception-action cycle in the brain and is critically involved in the most complex aspects of the selection, ordering and sequencing of behavioral and cognitive processes. In humans, three principal subdivisions of the PFC can be distinguished, namely orbital, medial and dorsolateral. These can be further characterized by specific glutamatergic inputs from different segments of the mediodorsal thalamic nucleus, a rich dopaminergic input mainly originating from the ventral tegmental area, along with specific outputs to different regions of the basal ganglia. Furthermore, the PFC can be also characterized by a relative lack of direct inputs from primary sensory areas, and direct outputs to the primary motor cortex. It possesses a unique pattern of supramodal connectivity with higher order cortical association areas and limbic structures such as the amygdala and hippocampus, as well as rich interconnections with the pre-motor areas. One of the mechanisms underlying its higher integrative functions may be the capacity of the PFC to retain short-term memory traces on the basis of information from the external world (concept of “working memory”). The guidance of behavior by the PFC is also critically dependent upon long-term memory traces stored in other parts of the brain and made available through the wealth of its limbic connections (concept of “working with memory”). Both working memory and long-term memory depend on a balanced interplay between glutamatergic and dopaminergic mechanisms.

## ABSTRACT-2

**Two faces of frontotemporal lobar degeneration: behavioral variant of frontotemporal degeneration and primary progressive aphasia**

M-Marsel Mesulam

Department of Neurology and Psychiatry, Northwestern University Medical Center, Chicago, Illinois.

Dementias can be classified as amnestic, comportmental or aphasic, according to the nature of the major impairment. Alzheimer disease typically leads to an amnestic dementia where memory loss is the major cause of impaired daily living activities. This is consistent with the hippocampal/entorhinal location of the initial neurodegeneration. The frontotemporal Lobar Degenerations (FTLD) constitute the second major class of dementias. The neuropathology is characterized by focal neuronal loss, gliosis, tau inclusions, and ubiquinated TDP-43 inclusions. Some forms of FTLD lead to combinations of motor and cognitive impairments as in CBD, PSP and FTD-MND. In other cases, FTLD leads to pure cognitive changes as in primary progressive aphasia (PPA) and the behavioral variant of frontotemporal dementia (bvFTD). Patients with bvFTD have preserved language and memory function but display major impairments of insight, judgment, working memory, problem solving and other executive functions. Disinhibition in the areas of sexual misconduct, shop lifting, impulsive gambling are frequently seen and fail to elicit remorse. The major atrophy in these patients is seen in prefrontal cortex, caudate nucleus and the temporal poles. The principal focus of this talk will be PPA, a focal neurodegenerative syndrome characterized by an isolated and gradual dissolution of word finding and word usage. The language disturbance is initially the most salient deficit and the major obstacle to the execution of daily living activities. This does not mean that there are no deficits other than the aphasia, but that such additional deficits are relatively minor in the first two years following symptom onset. Some patients develop prominent agrammatism, others profound word comprehension (semantic) deficits. The speech output in PPA can be fluent or non-fluent. Memory, visual processing and personality remain relatively preserved during the initial stages and help to distiguish PPA from bvFTD and the typical forms of AD. Terms such as progressive non fluent aphasia (PNFA) and semantic dementia (SD) have been used to denote subtypes of PPA. Structural and physiological neuroimaging confirms the selective predilection of PPA for language-related cortices. The majority of the autopsies in PPA have shown the neuropathology of FTLD. Recently two kindreds with familial PPA have been described and shown to be related to progranulin *PGRN* gene mutations on chromosome 17. ApoE genotyping and prion protein polymorphisms differentiate PPA from AD. The mechanisms that determine the initial selectivity of the cognitive impairment and the asymmetry of atrophy in PPA remain to be elucidated. An informed approach to PPA helps to address the challenges associated with the care of these patients. This syndrome also offers unique opportunities for exploring the cognitive architecture of language processing and the neurobiological fingerprints of the language network.

## ABSTRACT-3

**Neurodegenerative diseases characterized by predominant language impairment: Brazilian study of 53 patients**

^1^Mirna Lie Hosogi Senaha, ^2^Paulo Caramelli, ^1^Claudia Sellitto Porto, ^1^Ricardo Nitrini

Behavioral and Cognitive Neurology Unit, Department of Neurology, and Conitive Disorders Reference Center (CEREDIC). Hospital das Clínicas of the University of São Paulo School of Medicine, São Paulo, Brazil. Behavioral and Cognitive Neurology Unit, Faculty of Medicine, Federal Univesity of Minas Gerais.

Following the seminal paper by Mesulam (1982), there has been great interest in predominant language impairment in degenerative diseases.

### Objective:

To describe cases with progressive language disturbances.

### Methods:

53 patients with neurodegenerative disease and predominant language impairment were evaluated.

### Results:

Mean age (n=53; 32 women) was 70.2 years (±8.3). Symptoms began in the presenile phase in 23 patients (43.4%); 39 patients had fluent (73.6%) and 14 non-fluent aphasia. Of the 39 fluent patients, 19 presented semantic dementia (SD), 4 anomic aphasia, 2 “Wernicke aphasia”, 3 “pure verbal deafness” and 11 were not classifiable. The SD group showed preservation of syntactic and phonological aspects of language, as well as unimpaired comprehension of complex sentences. Surface dyslexia was diagnosed in 47.4% of the SD patients; semantic dyslexia in 21.0% and surface dysgraphia in 84.2%. In the non-fluent group, syntactic impairment occurred in 9 patients (64.3%) and dysartria or speech apraxia in 9 (64.3%). None of the non-fluent patients had surface dyslexia, but 28.5% presented surface dysgraphia. The SD group had lower performance than the non-fluent group in naming tasks. The SD category was more impaired on letter fluency, while the reverse was observed in the non-fluent patients. The 11 unclassifiable patients had overlapping characteristics of both SD and non-fluent groups.

#### Conclusions:

In our sample, fluent cases were more frequent than non-fluent; the age of onset was higher and there were more women than other series in the literature. More than 50% of the fluent patients were not classified as SD, and 28.2% were unclassifiable.

## ABSTRACT-4

**Human fronto-temporo-limbic circuits underlying moral cognition and altruism**

Jorge Moll^1^, Ricardo de Oliveira Souza^2^

^1^Cognitive Neuroscience Section, National Institute of neurological Disorders and Stroke, NIH, Bethesda, USA.

^2^Service of Internal Medicine, Graffrée and Guinle University Hospital, Rio de Janeiro; LABS-D’Or Hospitals Network, Rio de Janeiro.

Functional imaging and clinical evidence point to the role of a remarkably consistent network of brain regions in human moral cognition, including moral judgments and moral sentiments. We will review these scientific lines of evidence, and discuss a recently devised neuroscientific model of how culturally-shaped social knowledge and basic motivational states can be integrated to better understand the complex links among motivation, cognition and moral values. Furthermore, recent experimental evidence indicating differential roles of orbitofrontal (OFC) and limbic circuits in altruistic decisions guided by moral values, along with the functional relationships of these circuits to social attachment and human affiliative behaviors and moral aversion, will also be presented. These findings suggest that human morality and altruism emerge from the integration of prospective evaluations, provided by the evolutionarily novel anterior OFC, conceptual social knowledge, stored in the anterior temporal lobes, together with primitive mechanisms of social attachment and aversion dependent on limbic-basal forebrain structures.

## ABSTRACT-5

**New developments in clinical assessment of neurodegenerative dementia**

Sandra Weintraub

Division of Psychology; Cognitive Neurology and Alzheimer Disease Center; Northwestern Medical Faculty Foundation, Chicago, Illinois.

The most common form of dementia, Alzheimer disease (AD), is characterized by primary amnesia and additional cognitive deficits. The early memory loss of AD has a profound impact on activities of daily living (ADL) and limits independence even before other cognitive deficits become apparent. Memory loss, however, is not symptomatic of other neurodegenerative forms of dementia, especially those caused by frontotemporal lobar degeneration (FTLD). Instead, the onset of FTLD is marked by one of two major classes of non amnestic symptoms: primary progressive aphasia (PPA) and progressive behavioral and comportmental symptoms, also known as behavioral variant frontotemporal dementia (bvFTD). In these two FTLD syndromes, ADL are affected not by forgetfulness but rather by aphasia and reduced judgment/comportment/executive functions, respectively. Currently used measures of dementia severity, such as the Clinical Dementia Rating (CDR) have been based on the symptoms associated with AD. As a result, they don’t adequately sample the aphasic and behavioral symptoms relevant to PPA and bvFTD and could inaccurately represent the severity of functional impairment in these disorders. Thus, there is a need for clinical measures of dementia severity that can identify daily functional impediments in all forms of dementia, related to their very distinctive symptom profiles. In this presentation, new methods for assessing dementia will be discussed. First, the experience of the Uniform Data Set of the Alzheimer Disease Centers programs of the National Institute on Aging in the US will be described, emphasizing the identification of the salient impairment in each clinically unique form of dementia (Morris et al, 2006). Secondly, two ways to gauge dementia severity in FTLD will be presented. The first, the Activities of Daily Living Questionnaire (ADLQ; Johnson et al, 2004), is a caregiver-completed measure of functional capacity in patients with dementia. It shows different patterns of functional loss depending on the nature of the prominent cognitive and behavioral deficits in PPA, bvFTD and AD (Wicklund et al 2007). Patients with PPA are more functionally independent than patients with bvFTD of similar disease duration due to pervasive attention, executive function, and social competence deficits characteristic of bvFTD. The second approach to characterizing severity in dementia in a variety of dementing disorders describes a new modification of the Clinical Dementia Rating (CDR) (Berg et al, 1987; Morris et al, 1989). The CDR was originally designed to differentiate early AD from normal aging and to stage different levels of severity in AD. The modified version of the CDR targets symptoms associated with PPA and bvFTD. Preliminary data show that it may be more accurate in staging the level of severity in PPA and also in FTD than the original CDR. The practical relevance of these approaches to the clinical characterization of dementia will be discussed.

## ABSTRACT-6

**Theory of the mind and frontal lobes: controversies**

Benito Pereira Damasceno

Department of Neurology, Medical School, State University of Campinas - SP, Brazil.

The Theory of Mind (ToM) is the ability to infer the mental states, thoughts and feelings of others. Most studies have concluded that ToM is subserved by a functional brain system including temporo-parietal junction, temporal pole, amygdala, orbitofrontal cortex and, in particular, the medial frontal lobes. PET and fMRI studies of normal subjects have also detected functional subdivisions in the medial prefrontal cortex (mPFC), with the anterior-rostral mPFC being activated by ToM tasks. In the majority of neuropsychological studies, patients with frontotemporal degeneration (FTD) or delimited frontal lesions were impaired on typical ToM tests such as first- and second-order false belief and faux pas detection. These studies are, however, limited by a lack of precise lesion location, and most patients had suffered head trauma, associated with rather diffuse brain damage. Moreover, there are well documented cases with extensive bilateral prefrontal lesions which do not impair performance on ToM tasks, such as the GT case reported by Bird et al. (2004), with bilateral mPFC lesion, as well as several of our bifrontal and FTD cases. These negative findings seem to suggest that the medial frontal regions are not necessary or critical for ToM tasks. Thus, it may be the case that (1) ToM, as a high level complex mental function (consciousness of self and other), requires such a widespread brain network that other parts, in connection with intact frontal regions, are sufficient for its functional integrity; (2) there is a post-lesional recovery of function; or (2) the ToM tasks employed do not evaluate as intended.

## ABSTRACT-7

**Update on frontotemporal lobar degeneration neuropathology in the light of detection of TDP-43paties and progranulin mutations: insights of the new consensus**

Lea Tenenholz Grinberg

Department of Pathology, University of São Paulo School of Medicine, Brain Bank of the Brazilian Aging Brain Study Group; Albert Einstein Research Institute, São Paulo, Brazil

The frontotemporal lobar degenerations (FTLDs) are the neuropathological counterpart of frontotemporal dementia and comprise a heterogeneous group of neurodegenerative disorders. Recent discoveries in molecular genetics, biochemistry, and neuropathology of FTLDs have changed our understanding of this previously enigmatic group of diseases. The spectrum of the FTLDs continues to expand, making these entities confusing even for those familiar with the subject. In 2006, new insights have come to light. Firstly, TAR DNA-binding protein 43 (TDP-43) was recognized as the main component of the inclusions found in FTLD-U (ubiquitin-positive and tau-negative inclusions) with or without motor neuron disease, creating the TDP-43paties, the most frequent group of FTLDs. Secondly, *progranulin* (PGRN) gene mutations on chromosome 17q21 emerged as an important cause of familial FTLD-U. Following the recent insights about the selective vulnerability and different rates of progression of the presently known FTLD subtypes, additional research into the mechanism of neurodegeneration associated with them is both feasible and desirable. Based on these recent findings, an international group of specialists revised the neuropathological criteria of FTLD in 2007. The aim of the present work is to address the current neuropathological criteria of FTLD published in June 2007, and to explain them in an illustrative and schematic manner in order to provide the basis for a better understanding of results related to FTLD published to date, as well as findings of the audience.

## ABSTRACT-8

**Misdiagnosis in FTD patients before evaluation by specialists: variables associated to misdiagnosis**

Márcia Lorena Fagundes Chaves, Ana Luiza Camozzato, Renata Kochhann, Claudia Godinho, Letícia Forster

Medical Sciences Post-Graduation Course, Universidade Federal do Rio Grande do Sul School of Medicine, Porto Alegre, Brazil. Alzheimer Disease Center and Neurogeriatric Clinic from the Neurology Service of Hospital de Clinicas de Porto Alegre.

Of all dementia patients seen in the Neurogeriatric Clinic from 1999 onwards, only 6 were FTD. The importance of variables associated to misdiagnosis and decision-making for tertiary clinic evaluation is critical.

### Objective:

To hypothesize which variables could be associated to misdiagnosis, considering the small size of the FTD sample

### Methods:

6 FTD patients, 10 patients evaluated for memory complaints/suspicion of dementia, and 10 suspected Alzheimer disease cases seen in the same period were investigated. Samples were balanced for entry criteria into the study for the period, limiting to “typical” cases in each category after expert evaluation. Variables: cause of referral, earlier diagnosis, previous behavioral and cognitive symptoms, Mini Mental State Exam, memory and other domains at expert evaluation, time of disease and outcome were considered. Parametric data were analyzed by one-way ANOVA and Bonferroni *post hoc*, while the association test with the Fisher exact test were carried out for categorical variables.

### Results:

All AD, 90% of the suspected dementia group and 33% of FTD were referred to the specialist due to memory complaints (p=0.007). Most AD (80%) and few FTD (17%) had no previous diagnosis, while 1/3 of FTD (33%) and few AD (17%) were treated as depressed (p=0.036). Most AD (70%) and suspected dementia cases (90%) reported memory symptoms during the expert evaluation, while only 33% of the FTD subjects did so (p=0.003). A total of 33% of the FTD reported oral communication problems. All AD and FTD patients presented below-cutoff scores on the MMSE while all patients from the suspected dementia group were above the cutoff (p=0.0001). Duration of disease since diagnosis was similar between AD and FTD.

### Conclusion:

The most consistent cause of referral to the specialist was memory complaints. Among AD, most patients did not present earlier diagnosis, while among FTD both depression and Alzheimer disease were frequent, memory complaints and behavioral changes being the most common causes of referral. Detection of behavioral symptoms suggests that these might influence the way clinicians tend to deal with these conditions and may affect the search for specific evaluations.

## ABSTRACT-9

**Novel symptoms in frontotemporal dementia**

Leonardo Caixeta

Adjunct Professor of Neuroscience, Federal University of Goiás (UFG). Coordinator, Cognitive and Behavioral Neurology Unit, Hospital das Clínicas-UFG.

We aimed to present two novel symptoms probably related to frontal lobe pathology.

### Methods:

We tested 20 patients with Frontotemporal dementia (FTD) and 20 patients with Alzheimer disease (AD) in order to elicit these behaviors. Objects such as papers of different sizes and shapes, and different colored pens as well as wood blocks of different sizes and colors (from the Token Task) were placed on the table in a random arrangement within subjects’ visual field.

### Results/Discussion:

Only 4 patients (20%) from the AD group presented such behaviors versus 16 (80%) from the FTD group. We called the first “ordenate behavior”, characterized by a compulsion to ordenate, in a very obsessive manner, objects placed in front of the patient on the table, without having been given any instructions to manipulate them (and even in the presence of a warning from the examiner not to touch the objects). A characteristic of such behavior is that the patient tends to “organize” material, placing previously spread papers into a rigid order, stacking material by paper size, with larger papers under smaller ones, while aligning the corners. The second behavior consists of a compulsion to group things placed in their visual field using some sort of category (for example, when presented with different colored pens as well as wood blocks of different sizes and colors, from the token task). A characteristic of such behavior is that patients tend to cluster objects according to their color, size, shape, etc.

## ABSTRACT-10

**Behavioral and activities of daily living inventories in frontotemporal lobar degeneration and Alzheimer disease – A pilot study**

Valéria Santoro Bahia, René Viana, Jerusa Smid, Antonio Eduardo Damin, Mari Nilva Maia da Silva, Márcia Radanovic, Ricardo Nitrini

Behavioral and Cognitive Neurology Unit, Department of Neurology, and Conitive Disorders Reference Center (CEREDIC). Hospital das Clínicas of the University of São Paulo School of Medicine, São Paulo, Brazil.

The differential diagnosis between frontotemporal lobar degeneration (FTLD) and Alzheimer disease (AD) can sometimes be challenging.

### Objectives:

To verify the utilility of behavioral and activities of daily living inventories in the differential diagnosis between FTLD and AD.

### Methods:

Caregivers of 10 patients with FTLD and of 10 patients with probable AD were interviewed. The mean MMSE score was 12.2±11.2 for patients with FTLD and 9.8±6.7 for patients with AD (p=0.89). The Frontal Behavioral Inventory (FBI), Neuropsychiatry Inventory – Q (NPI-Q) and Disability Assessment for Dementia (DAD) were utilized.

### Results:

Mean scores on the DAD were 37.8± 29.0 in patients with FTLD and 54.3±28.4 in patients with AD (p=0.15), while on the FBI were 42.6±10.5 for FTLD and 20.3±12.4 for AD (p=0.01), and on the NPI-Q, mean scores were 12.2±11.2 for FTLD and 9.8±6.7 for AD (p=0.45).

### Conclusion:

In this initial assessment, FBI was the best inventory for differential diagnosis between FTLD and AD.

## ABSTRACT-11

**Working memory in Alzheimer disease**

Cybelle Diniz, Paulo Hentique Ferreira Bertolucci

Behavioral Neurology Section – Escola Paulista de Medicina – UNIFESP

### Objective:

Memory deficit is a major impairment in AD, but despite the large body of research on explicit memory, less attention has been given to working memory. Considering that executive deficits are often present at an early stage, this warrants an important issue for investigation. The present study investigated working memory in AD as part of a more comprehensive project investigating executive function.

### Methods:

23 probable AD subjects (age – 72.9±7.0; education – 7.9±4.0; MMSE – 25.2±1.2) were compared with 33 control subjects (age – 70.4±5.8; education – 9.2±4.4; MMSE – 28.5±1.4). For the evaluation of working memory the Brown Peterson Paradigm (BPP) was used.

### Results:

BPP scores showed a significant difference between AD (6.6±2.0) and CG (10.4±4.4) (p<0.001). There was no effect of gender on performance in the test, but an influence of age (p=0.001) and education (p=0.009) was observed. There was significant correlation between BPP and Neuropsychiatric Inventory scores (p=0.031), besides a near significant correlation between BPP and functional capacity (p=0.073).

### Conclusions:

deficits in working memory may be present at an early stage of AD where such deficits may have a significant impact on relevant aspects of daily living. This is an area that warrants further attention.

## ABSTRACT-12

**Pharmacological treatment of frontotemporal lobar degeneration systematic review**

Valeska Marinho^1^, Maria da Glória Portugal^2^, Eliasz Engelhardt^3^, Jerson Laks^4^

^1^Doctorate in Sciences EPM/UNIFESP, Physician at Center for Alzheimer Disease – Psychiatry Institute, Federal University of Rio de Janeiro, Brazil.

^2^Physician Psychiatrist.

^3^Cognitive and Behavioral Neurology Section – Neurology Institute, Federal University of Rio de Janeiro, Brazil.

^4^Coordinator of the Center for Alzheimer Disease- Psychiatry Institute of the Federal University of Rio de Janeiro; Adjunct Professor of the Medical Sciences Faculty, Rio de Janeiro State University.

### Objective:

To identify therapeutic options available for the treatment of cognitive and behavioral symptoms of Fronto-Temporal Lobar Degeneration (FTLD).

### Methods:

Systematic Review. Electronic Data Bases: MEDLINE, LILACS and Biological Abstracts; manual search among cited references and leading specialized journals. Descriptors: frontotemporal lobar degeneration OR frontotemporal dementia OR fronto-temporal dementia OR fronto-temporal degeneration OR Pick´s disease OR Pick's atrophy OR semantic dementia OR progressive aphasia AND pharmacotherapy OR treatment OR efficacy OR effects OR management. Selection Criteria: Quality A- Randomized, placebo-controlled and double-blind clinical studies. Quality B- Open studies or reports on six or more cases. Quality C- Reports of five cases or less. Review articles were excluded. Information collection: two reviewers independently assessed the clinical studies. Information collected included diagnosis criteria employed, sample size, duration, efficacy and tolerability measures used and results obtained.

### Results:

A total of 355 studies were found, 15 of which were selected for analysis. All studies involved small samples, had short treatment periods, and employed non-uniform measures for efficacy and tolerability assessment. Results for behavior and cognition differed amongst studies.

### Conclusion:

Evidence available for treatment of FTLD indicates that serotonin reuptake inhibitors are the most used substances. However, none of the compound classes had demonstrated consistent benefits regarding cognitive and behavioral aspects of FTLD.

## ABSTRACT-13

**Symptoms of obsessive-compulsive spectrum in frontal lobe lesions**

Francisco de Assis Carvalho do Vale, Pena MCS, Sobreira EST, Pitelli C, Paccola ATF, Schmidek CM

Behavioral Neurology Group, Clinics Hospital of the Ribeirão Preto Faculty of Medicine.

Frontal lobe lesions usually cause behavioral disturbances and personality changes, with relative preservation of memory, language, and other cognitive functions. The impairment of frontal corticocortical, corticobasal thalamocortical circuitries and their connections with the limbic system may produce symptoms of the obsessive-compulsive spectrum (SOCS). Such symptoms are characterized by intrusive, reverberative thoughts which generate compulsive, repetitive behaviors, as occurs in obsessive-compulsive disorder.

### Objective:

Identify SOCS in patients with frontal lobe lesions followed at a tertiary care outpatient clinic of a teaching hospital.

### Method:

We took 89 (5.5%) cases of individuals with frontal lobe lesions from an 11-year casuistry of the Behavioral Neurology Outpatient Clinic, Clinics Hospital of the Ribeirão Preto Faculty of Medicine. We assessed 40 (44.9%) of these patients through a clinical interview emphasizing the active search for SOCS. These patients had age ranging from 17 to 77 years and 70.0% were male gender.

### Results:

The most frequent causes were frontotemporal dementia (FTD) (32.0%) and head trauma (32.0%). Other causes were vascular lesion (5 cases), Alzheimer disease (3), Lewy body dementia (1), arachnoid cyst (1), meningoencephalitis (1), lobotomy (1), multiple system atrophy (1) and mucormycosis (1). Eight (20.0%) of the assessed patients had SOCS. SOCS were diverse, including intrusive thoughts, rituals and repetitions. Four of the SOCS patients had suffered head trauma, one had FTD, one had an arachnoid cyst, one had AD and one had multiple system atrophy.

### Conclusions:

SOCS may be under-diagnosed in patients with frontal lobe lesions. The great diversity of SOCS found in such a small casuistry highlights the complexity of the frontal cortical and subcortical networks involved.

## ABSTRACT-14

**Case report: Frontotemporal dementia: clinical, neuropsychological and neuroimaging findings**

Sérgio Ricardo Hototian, Renata Ávila Psych, Dionísio de Azevedo, Cândida Pires de Camargo, Cássio Machado de Campos Bottino

Old Age Research Group (PROTER), Institute and Department of Psychiatry, University of São Paulo School of Medicine, São Paulo, Brazil.

A seventy-year-old female patient was referred to the Outpatient Service of the Old Age Research Project (PROTER), Institute and Department of Psychiatry, University of São Paulo School of Medicine, with suspected diagnosis of “Dementia Syndrome” with one-year onset. Patient “A” was brought by her family and presented a clinical history of three years of aphasia, followed by behavioral change, and slight memory deficit for recent events as the last and most recent symptom. A year before the first medical consultation in PROTER, the patient had started presenting dementia. These alterations had started interfering with her activities of daily living and decreased her occupational functioning, albeit slightly. The results of a first evaluation, composed of neuropsychological testing, clinical examination, history data and complementary exams, led to the diagnosis of mild to moderate Frontotemporal Dementia. After attending the medical consultations in PROTER over a one-year period, the patient evolved to severe dementia, from MMSE score of 18 to 7. After 2 years and 7 months of follow up, the patient was completely dependent with 0 score on the MMSE, being clinically classified as having Progressive Frontotemporal Dementia.

## ABSTRACT-15

**Use of memantine for behavioral and cognitive symptoms in semantic dementia**

Maria da Glória Portugal^1^, Valeska Marinho^2^, Eliasz Engelhardt^3^, Jerson Laks^4^

^1^Psychiatrist.

^2^MD, PhD, Psychiatrist Center for Alzheimer Disease and Related Disorders - Institute of Psychiatry - Federal University of Rio de Janeiro, RJ, Brazil.

^3^MD, PhD, Head Professor, Section of Cognitive and Behavioural Neurology - Institute of Neurology - Federal University of Rio de Janeiro, RJ, Brazil.

^4^MD, PhD, Associate Professor State University of Rio de Janeiro, Center for Alzheimer Disease and Related Disorders - Institute of Psychiatry - Federal University of Rio de Janeiro, RJ, Brazil.

We report a case of a 55-year-old woman who came for evaluation due to impairment in naming. Language dysfunction began prior to the behavioral abnormalities, characterized as apathy, irritability, stereotyped behavior and decline in hygiene. Clinical assessment included psychiatric and neurological history and examinations, neuropsychological evaluation using MMSE and NPI. Brain MRI scan showed cortical atrophy with left frontotemporal predominance and right frontal sulci enlargement. Magnetic Resonance Spectroscopy of the frontal lobes showed decreased N-acetyl aspartate levels, and a slight increase of myo-inositol, while the posterior cingulate area showed increased myo-inositol levels without other biochemical abnormalities. Considering the clinical and neuroimaging data, a diagnosis of Semantic Dementia was reached, according to the Lund and Manchester Group diagnostic criteria. Memantine 10 mg/day was prescribed for one month, adjusted to 20 mg/day thereafter. Following 12 weeks of pharmacological treatment there was a clinical impression of slight improvement on behavioral and cognitive functions, also observed through clinical measures of efficacy such as MMSE and NPI scores, which were both slightly changed compared to baseline evaluations.

## ABSTRACT-16

**Neuropsychological differences between the performance of patients with frontotemporal lobar degeneration and Alzheimer disease**

Claudia Sellitto Porto, Valeria Santoro Bahia, Sônia Maria Dozzi Brucki , Paulo Caramelli, Ricardo Nitrini

Behavioral and Cognitive Neurology Unit, Department of Neurology, University of São Paulo School of Medicine; Cognitive Disturbances Reference Center, Hospital das Clínicas of the University of São Paulo School of Medicine; Department of Internal Medicine, Faculty of Medicine, Federal University of Minas Gerais, Belo Horizonte (MG).

### Objectives:

To investigate possible differences between the performance of patients with frontotemporal lobar degeneration (FTLD) and patients with Alzheimer disease (AD) in neuropsychological tests.

### Method:

Fifty-six AD patients (mean age=72.98±7.43; mean schooling=9.62±4.68; 35 women and 21 men) and 17 FTLD patients (mean age= 67.64±7.93; mean schooling=12.12±4.77; 9 women and 8 men) were submitted to a comprehensive neuropsychological evaluation composed of tasks assessing attention, visuoperceptual abilities, constructive abilities, executive functions, memory and language.

### Results:

FTLD and AD patients showed statistically significant differences in their performance on tests of verbal (Logical Memory, Rey Auditory Verbal Learning Test) and visual (Visual Reproduction, recall of the Rey Complex Figure) episodic memory, verbal immediate memory (Logical Memory) and attention with interference (Trail Making Test – Part B).

### Conclusion:

In contrast to expectations only one task of executive function (Trail Making Test – Part B) together with verbal and visual episodic memory tests, were able to differentiate FTLD patients from AD cases.

## ABSTRACT-17

**Woman with long-term bipolar disorder develops frontotemporal lobar degeneration**

Tibor Rilho Perroco, Renné P Alegria

Old Age Research Group (PROTER), Institute and Department of Psychiatry, University of São Paulo School of Medicine, São Paulo, Brazil.

### Case Report:

A 79 year-old woman with four-year educational level is described. Her daughter reported that her mother had a diagnosis of long-term Affective Bipolar Disorder. The patient’s clinical picture had always been one of very serious episodes of mania, and involved multiple involuntary institutionalizations. Her behavior changed markedly over the past year, becoming quieter, mainly during maniac episodes, standing alone while being dependent on others, She no longer visits the hairdresser on her own, has lost urinary sphincter control, leaves objects everywhere, displays aberrant motor behavior, needs help undressing, but reports no visual or auditory hallucinations. The subject has been institutionalized for at least four years; “She never likes where she is”. She has had four falls this year and complains of “limp and trembling” legs, pain in the lumbar region and difficulty in locomotion. Her assessment revealed MMSE: 15/30, apathy, difficulty in ambulation, psychomotor slowness. NMR image of the brain shows evident volumetric reduction of the frontal and temporal lobes bilaterally. The hippocampi had normal dimensions and there was hyperintensity of frontal white matter.

### Conclusion:

Patient with ABD and current diagnostic of FTLD. This case raises the need to investigate some questions: Can ABD evolve to FTLD? Can excessive use of medication play a role in this evolution? Are such patients at greater risk of developing a concomitant dementia?

## ABSTRACT-18

**Very early manifestations of frontotemporal dementia: the role of magnetic resonance imaging**

Andre Palmini, Luciana Tisser, Mirna Portuguez

Neurology Service, Hospital São Lucas da PUCRS. Porto Alegre, Brasil.

Fronto-temporal dementia (FTD), frontal lobe type, is typically characterized by the insidious onset of behavioral and personality abnormalities. Usually, however, these early manifestations are not accompanied by distinct structural imaging abnormalities, which only tend to present after considerable dysfunction is already evident.

### Objective:

To report a woman with very early manifestations of FTD in whom, atypically, MRI showed significant, bilateral frontal lobe abnormalities.

### Key clinical, neuropsychological and imaging findings:

At presentation, this 58 year-old woman was fully functioning socially, and worked daily, helping to run a commercial family business. She had started 2 years earlier with what was simply considered an exacerbation of her usually extroverted behavior. She was more ‘intense’ than usual when discussing with neighbors, strongly verbalized that she ‘hated’ particular individuals with whom she had argued, and acted inappropriately in social gatherings. A comprehensive neuropsychological evaluation showed normal verbal memory scores, yet significantly (>1 SD) reduced scores on the Wisconsin Card Sorting Test, semantic fluency (animals) and digit span. Fluid-attenuated inversion recovery (FLAIR) MR images showed increased signal and atrophy of the fronto-striatal pathways. No other significant abnormalities were seen. A diagnosis of f\FTD was then made.

### Conclusion:

In certain patients, very early manifestations of FTD may be accompanied by distinct MRI abnormalities, which seem discrepant with the mild clinical picture, but allow early diagnosis.

## ABSTRACT-19

**Increasing the assessment of frontotemporal dementia (FTD) in a Southern Brazilian University Hospital**

Analuiza Camozzato de Pádua e Márcia Lorena Fagundes Chaves

Neurology Service - Neurogeriatric and Dementia Clinic. Hospital de Clínicas de Porto Alegre (HCPA).

Few identified or suspected Fronto-Temporal Dementia (FTD) cases have been referred to the Neurogeriatric and Dementia Clinic of Hospital de Clínicas de Porto Alegre (HCPA) over the last 2 years. We hypothesized that more cases of FTD should be referred for evaluation within a university hospital. FTD patients could be erroneously diagnosed as depressive, bipolar or with other psychiatric disorders.

### Objective:

The study aimed to devise and validate a screening instrument in order to increase the detection of FTD in HCPA.

### Methods:

A short questionnaire answered by caregivers or physicians to screen for FTD based on behavioral and mood changes was developed by our research team. Concurrent validity with Midelheim Frontality Score (MFS) will be studied. The gold standard will be the Consensus diagnostic criteria for FTD. This study will be conducted in a Mood Disorder Clinic and Psychiatry Inpatient Unit within HCPA.

### Results:

This study is undergoing implementation and no results are yet available.

### Conclusions:

We trust this new instrument will be valid and improve FTD screening in our setting.

## ABSTRACT-20

**Alzheimer disease mimicking progressive primary aphasia: a case report**

Norberto Anísio Ferreira Frota^1^; Mari-Nilva Maia da Silva^2^

^1^Department of Neurology, University of São Paulo, postgraduate student.

^2^Department of Neurology, University of São Paulo, Behavioral and Cognitive Neurology Unit Fellow.

In October 2006, a 51-year-old previously healthy white woman, with 11 years of formal education and an employee in a medical research institute, was brought by her family to the cognitive neurology unit. She complained of difficulty in finding words during dialogue. Symptoms had started about two years earlier and insidiously worsened with disturbed reading and writing. More recently, she had started to lose objects and forget messages that people asked her to give. She had never got lost, but her family began to follow her, fearing that it could happen. In the first assessment she scored 13 in the mini mental state examination (MMSE), the worst score being in expressive language, with dysgraphia and constructive apraxia. She scored zero in the inverse digit span while in the brief battery of cognitive screening (BBRC) she was able to name all the 10 figures, registered 7 and retrieved 6 after distraction task. Also, in the task of visual agnosia she was unable to recognize 12 of the 18 figures, with a similar score in both concrete and abstract figures. The subject scored 2 in clock drawing, and presented ideomotor apraxia in bimanual gestures at neurological examination. In the complementary neuropsychological evaluation, she failed in memory and executive function, in addition to language. MRI showed diffuse cortical atrophy. SPECT showed moderately reduced blood flow in temporal and parietal lobes bilaterally. Infection and metabolic causes were ruled out. In this case, language disturbance was the first and only complaint for about two years. Memory was impaired in some tests but not in others. The diagnosis of Primary Progressive non-fluent Aphasia was suggested, but complementary assessment corroborated a diagnosis of Alzheimer disease.

## ABSTRACT-21

**Semantic memory impairment in temporal lobe epilepsy associated with MRI evidence of hippocampal sclerosis**

Cristiane Stravino Messas^1^; Letícia Lessa Mansur^1^; Luiz Henrique Martins Castro^2^

This study was performed in the Department of Physical, Speech and Occupational Therapies at the Medical School of University of São Paulo^1^; Hospital das Clínicas of the University of São Paulo School of Medicine, Brazil^2^.

Episodic memory impairment is a well-recognized feature of mesial temporal lobe epilepsy. Semantic memory has received much less attention in this patient population. We studied semantic memory aspects (word-figure matching, word definition, confrontation and responsive naming and word list generation) in 19 patients with left or right temporal lobe epilepsy, secondary to mesial temporal sclerosis (MTS), and compared them with normal controls. Patients with left MTS showed impaired performance in word definition, responsive naming and in word figure-matching, although this latter difference did not reach statistical significance. Both left and right left mesial temporal lobe patients performed worse than controls in word list generation and in confrontation naming tests. Attentional-executive dysfunction may have contributed to these deficits. We conclude that left MTS patients display impaired aspects of semantic knowledge. A better understanding of semantic processing difficulties in these patients will provide a deeper insight into the difficulties in performing activities of daily living seen in this patient population.

## ABSTRACT-22

**Applicability of the Arizona Battery for Communication Disorders in Dementia (ABCD) to evaluate language in patients with frontotemporal dementia: preliminary study**

Letícia Lessa Mansur, Pongrácz GM, Carvalho IAM, Valeria Santoro Bahia, Ricardo Nitrini

This study was performed in the Department of Physical, Speech and Occupational Therapies at the Medical School of University of São Paulo; Behavioral and Cognitive Neurology Unit, Department of Neurology, and Conitive Disorders Reference Center (CEREDIC). Hospital das Clínicas of the University of São Paulo School of Medicine, São Paulo, Brazil.

Frontal-temporal dementias (FTDs) result from focal brain damage and display clinical manifestations which differ from those verified in Alzheimer disease (AD), where episodic memory is the most predominant symptom. In FTDs, semantic memory deficit, language deficit and behavior disturbance stand out and are correlated to the impaired neural substrate. Currently, there is no specific language test to assess FTDs while the applicability of AD assessment in FDTs is not known.

### Objectives:

To analyze language in FTD cases and verify the applicability of the Arizona Battery Test to assess language in FTD.

### Method:

Eight female patients diagnosed with FTD, mostly with frontal alteration, aged from 45 to 63 years and with 4 to 15 years of education from the Cognitive and Behavioral Neurology Group of the Clinical Neurology Division of School of Medicine (FMUSP) participated in the study. The control group (CG) was composed of six subjects with no dementia, matched for age and education. All individuals from both groups were assessed with the Arizona Battery for Communication Disorders in Dementia (ABCD) (Bayles, 1994), adapted to Brazilian Portuguese, including subtests of mental state, episodic memory, language expression, language comprehension, and visual-spatial skills. The results were scored according to the ABCD protocol and group performances compared.

### Results:

FTD patients had lower average scores compared to controls in subtests related to mental state (CG=10.25; FTDg=12.50, p=0.042); language expression (CG=58.83; FTDg=26.75, p=0.002) and language comprehension (CG=94.67; FTDg=68.25, p= 0.012). No difference between groups was seen for episodic memory and visual-spatial subtests.

### Conclusion:

The results of this sample agree with the literature reporting greater language deficits than episodic memory deficits in FTD. The ABCD is a useful instrument for differential diagnosis between FTD and Alzheimer disease. 

## ABSTRACT-23

**Analysis of oral discourse in frontotemporal dementia**

Letícia Lessa Mansur, Carvalho IAM, Pongracz GM, Valeria Santoro Bahia, Ricardo Nitrini

This study was performed in the Department of Physical, Speech and Occupational Therapies at the Medical School of University of São Paulo; Behavioral and Cognitive Neurology Unit, Department of Neurology, and Conitive Disorders Reference Center (CEREDIC). Hospital das Clínicas of the University of São Paulo School of Medicine, São Paulo, Brazil.

Discourse analyses verify phonologic, syntactic, semantic and pragmatic levels of communication. Frontal-temporal dementia (FTD) patients may experience difficulties in any of these linguistic levels as a result of an inability to be aware of contexts, decide on relevance and integrate these elements according to such features. Oral discourse is organized by grammatical characteristics in which cohesion and coherence are determined by a sequence of communicative exchanges.

### Method:

Eight FTD patients (FDTG) aged from 45 to 63 years and with 4 to 15 years of education, along with a control group (CG) composed of six subjects with no dementia matched for age and education, were assessed using four boards numbered from one to four, of increasing visual complexity, on the composing of oral descriptive discourse for each board.

### Results:

The most marked errors found were *pauses longer than two seconds*, verified for all four boards, reaching statistical significance (p=0.003, p=0.029, p=0.002, p=0.002, respectively); while words substitution was statistically significant for board 1 (p=0.035), 3 (p=0.048) and 4 (p=0.004). There were also errors of *words revision* for board 1 (p=0.014), and *words repetition* (p=0.005), *higher use of functional words* (p=0.042) and higher number of *substantives* (p=0.024) for board 3.

### Conclusion:

The errors described above may be due to failure of both linguistic formulation and monitoring. It is important to highlight that patient discourse tasks improved through greater context.

### ABSTRACT-24

**Case of semantic dementia**

Letícia Lessa Mansur, Sonia Maria Dozzi Brucki, Cláudia Sellitto Porto

Behavioral and Cognitive Neurology Unit. Department of Neurology, University of São Paulo School of Medicine, São Paulo, Brazil. This study was performed in the Department of Physical, Speech and Occupational Therapies at the Medical School of University of São Paulo.

We describe the case o f a 61 year-old woman with 4 years of schooling, who came to the hospital with a complaint of: *“I forget names but I know everything”*. Her husband reported that about 2 years earlier, his wife has begun to progressively forget names of objects and persons. Presently she becomes confused when preparing food, miscalculating condiments and cooking time, washes up inadequately and shows mental inflexibility and rigid thoughts. In addition, she cannot understand what she has watched on TV. Her evaluation revealed a normal neurological examination, except for cognitive alterations. She brought a CT brain scan showing bi-temporal atrophy, with severe compromise on the left side. Her cognitive examination revealed: MMSE of 13; clock design: 2; verbal fluency (animals): 0. Her global cognitive status was evaluated by the Dementia Rating Scale (DRS) which yielded a poor total score (37/144), with greatest impairment in the Conceptualization subscale. Her constructive abilities were relatively preserved in contrast to the other cognitive functions, such as attention, memory and visual perception. She was able to recognize objects involved in her daily life activities, yet demonstrated severe compromise in recognition tasks involving living things. There were no complaints regarding recognition of faces or sounds. In the language examination, she was unable to recall the name of stimuli (floor effect) and did not recognize visual representations of a large number of them. Additionally, surface dyslexia was noted in written language. Discrepancies in semantic knowledge together with the best approach in language functions shall be discussed for this case.

## ABSTRACT-25 

**Analysis of FTD patient performance in specific category naming tests**

Letícia Lessa Mansur, Pongracz GM, Carvalho IAM, Valeria Santoro Bahia, Ricardo Nitrini

This study was performed in the Department of Physical, Speech and Occupational Therapies at the Medical School of University of São Paulo; Behavioral and Cognitive Neurology Unit, Department of Neurology, and Conitive Disorders Reference Center (CEREDIC). Hospital das Clínicas of the University of São Paulo School of Medicine, São Paulo, Brazil.

Language problems are frequent in frontal-temporal dementias (FTDs). On behavioral manifestation, attentional support systems account for the linguistic deficit and may cause difficulties in pseudo-word processing, whereas for semantic manifestation, semantic memory deficits predominate and may impact the processing of animate and inanimate categories.

### Objective:

To verify the performance of FTD patients in specific category naming tests.

### Method:

Eight patients (FDTG), mostly with frontal alteration, aged from 45 to 63 years and with 4 to 15 years of education from the Cognitive and Behavioral Neurology Group of the Clinical Neurology, participated in the study. The control group (CG) was composed of six subjects with no dementia, matched for age and education. Naming tests of specific categories of instruments, transports, animals, food, body parts, utensils, clothing, names of famous people and well-known places were applied.

### Results:

All categories except name of transport and clothing were shown to be lower for patients than controls (instruments: CG=6, FTDG=3, p=0.001); animals: CG=26.67, FTDG=16, p=0.019); food: CG=11.17, FTDG=6, p=0.002; body parts: CG=6, FTDG=5.67, p=0.034; utensils: CG=23.33, FTDG=14.88, p=0.005; personalities: CG=19.50, FTDG=10.38, p=0.002; places: CG=11, FTDG=4.13, p=0.004).

### Conclusion:

The semantic deficits were equally noted in naming of animate and inanimate categories as well as in personalities and places. In this sample, semantic deficits may show lexical access difficulties and not deterioration of meaning. Qualitative analysis of errors is necessary to make this distinction.

## ABSTRACT-26

**Planning and strategy application disorders in frontal lobe lesion patients**

Paula Adriana Rodrigues Gouveia^1^, Sonia Maria Dozzi Brucki^2^, Suzana Maria Fleury Malheiros^1^, Orlando Francisco Amodeo Bueno^1^

^1^Universidade Federal de São Paulo (UNIFESP), São Paulo, SP, Brazil. ^2^Hospital Santa Marcelina, São Paulo, SP, Brazil.

### Abstract:

The aim of this study was to investigate deficits in planning ability using an adapted version of the Modified Six Elements Test, from the Behavioral Assessment of the Dysexecutive Syndrome-BADS [Wilson, B. A., Alderman, N., Burgess, P. W., Emslie, H., & Evans, J. J. (1996). *Behavioural Assessment of the Dysexecutive Syndrome* (BADS). Bury St Edmunds, U.K.: Thames Valley Test Company. Trans. Ricardo O Souza, Sergio L Schmidt. Rio de Janeiro: Cognição]. Subjects were left- and right-frontal lobe lesion patients. Other measures of executive dysfunctions used were verbal Fluency, the Wisconsin Card Sorting Test, and the Trail Making Test. These other instruments were sensitive in detecting executive deficits in the left frontal lobe lesion group, except the Wisconsin Card Sorting Test, which detected impairment only for the frontal lobe lesion group as a whole. The Modified Six Elements Test detected planning disorders in left frontal lobe lesion patients. The deficit of these patients was due to a greater likelihood of breaking the rules of the task, that is, in plan-following processes, rather than in planning the strategic approach to solve it.

## ABSTRACT-27

**Cognition and depressive symptoms in a community sample of elderly subjects from São Paulo, Brazil**

Ricardo Barcelos, Jéferson Cunha Folquitto, Sergio Barbosa Barros, Litvoc Julio, Cassio Machado de Campos Bottino

Old Age Research Group (Proter). Institute of Psychiatry – University of São Paulo Medical School – São Paulo – Brazil.

Depression is a serious health problem in the elderly (Rinaldi et al., 2003), and perhaps is the most frequent cause of emotional suffering and impairment in quality of life (Blazer et al., 1991). Depression in clinical samples affects around 5% to 10% of outpatients and 9% to 16% of inpatients, generating increased mortality and costs of treatment (Katon, 2003; Cooper et al., 2002). A recent meta-analysis showed that persons with a history of depression were more likely to be diagnosed as having Alzheimer disease later in life (Ownby et al., 2006).

### Objective:

To determine the frequency of depressive symptoms in a population sample of elderly Brazilians and their association with social-demographic factors, cognitive deficits and other clinical and psychiatric conditions.

### Methods:

A randomized population sample of 1,563 individuals was evaluated through a screening scale of depression for the elderly with 10 items (GDS-5+5 items), the Mini-Mental State Examination (MMSE), Bayer Activities of Daily Living scale (B-ADL) and a social-economic questionnaire.

### Results:

The frequency of depressive symptoms varied from 16.1% to 61.6%. Multivariate analysis revealed association of these symptoms with B-ADL (B=0.057; p<0.001), previous history of depression (B=0.223; p<0.001), psychotropic use (B=0.217; p<0.001), physical exercises (B= –0.163; p< 0.001), hypertension (B=0.127; p<0.001), diabetes (B= 0.105; p<0.015) and holding a job (B=0.104; p<0.003).

### Conclusion:

There was a high frequency of depressive symptoms along with significant association with activities of daily living, and other clinical conditions such as diabetes and hypertension. In this cross-sectional survey, we did not observe any association of depressive symptoms with lower MMSE scores.

## ABSTRACT-28

**Functional communication ability in frontotemporal dementia and Alzheimer disease**

Carvalho IAM, Letícia Lessa Mansur, Valeria Santoro Bahia, Ricardo Nitrini

This study was performed in the Department of Physical, Speech and Occupational Therapies at the Medical School of University of São Paulo; Behavioral and Cognitive Neurology Unit, Department of Neurology, and Conitive Disorders Reference Center (CEREDIC). Hospital das Clínicas of the University of São Paulo School of Medicine, São Paulo, Brazil.

Functional communication is crucial for independent and efficient communicative behavior in response to every day activities. As dementia progresses, cognitive losses may impair these abilities. For this reason, functional communication assessment should be part of a formal assessment to quantify and qualify the impact of the deficiency on patient life.

### Objective:

to compare functional communication abilities in frontal-temporal dementia (FTD) and Alzheimer disease (AD).

### Methods:

Six AD patients (mean age: 82.50± 2.66; mean education: 5.67±3.61 years) and eight FTD patients (mean age: 57.13±9.63; mean education: 10.86±6.91 years) had their close relatives answer the Functional Assessment of Communication Skills for adults (Asha-facs). Statistical analyses correlated the performance on each of the Asha-facs domains (social communication, communication of basic needs; reading, writing, number concept and daily planning) between both groups.

### Results:

Analyses showed that functional communication was similar for AD and FTD patients. Only two items revealed statistical difference, namely, ‘Comprehension of inference’ (AD 6.7±1.33; FTD 2.43±2.30, p=0.017) and ‘capacity to make basic money transactions’ (AD 2.17±2.04; FTD 4.00±0.90, p=0.044). Comparison of mean scores for the four domains revealed no significant difference. Conclusion: Indirect assessment with family members or caregivers through a functional evaluation scale predicts deficits and abilities in an ecological analysis, and minimizes patient exposure to long and exhausting cognitive testing. Although a deficit of functional communication ability in both AD and FTD is known to exist, the analysis presented using our small sample showed that the Asha-facs could not discriminate between FTD and AD on these aspects.

## ABSTRACT-29

**Primary progressive aprosodia: a right variant form of primary progressive aphasia?**

Leonardo Caixeta^1^, Lea T. Grimberg^2^, Sérgio Rosemberg^2^, Wesley Silva^3^, Élbio de Paula^3^

^1^Adjunct Professor of Neuroscience, Federal University of Goiás (UFG). Coordinator, Cognitive and Behavioral Neurology Unit, Hospital das Clínicas-UFG.

^2^Department of Pathology of Faculty of Medical Sciences of University of Sao Paulo.

^3^Department of Pathology, Faculty of Medical Sciences, Federal University of Goias.

Primary progressive non-fluent aphasia (PPA), described in 1982 by Mesulan, was regarded as the clinical phenotype when left frontal-temporal degeneration predominated early on in the disease. Since then, several authors have drawn attention to the fact that the features of primary progressive aphasia were frequently atypical according to traditional classification of aphasia. These reports do not describe a sole clinical syndrome. Several patterns of deficit have been encompassed by the broad rubric of “progressive aphasia”, reflecting breakdown in distinct functional systems. We describe a patient with focal right anterior atrophy presenting clinical slowly progressive speech production deficit combining speech apraxia, dysarthria, dysprosody and orofacial apraxia, together with no other initial deficit in other language and non-language neuropsychological domains. CT and MRI findings disclosed asymmetric (right>left) progressive cortical atrophy of the right hemisphere, predominating in the posterior inferior frontal region, notably the operculum. SPECT revealed asymmetric decreased cerebral blood flow and metabolism, prominent in the right posterior-inferior frontal gyrus and premotor cortex. Anatomo-pathological study was performed. This case extends the clinical spectrum of focal cortical syndromes to include a primary progressive degeneration of the right hemisphere, with distinct clinical and neuroimaging presentation of PPA, while disclosing clinical features of a right hemisphere language disorder with dysprosodia and apragmatism. In the light of this, the term PPA may not be appropriate to encompass all phenotypes of focal cortical syndrome related with language disorders. Perhaps in pathological terms, Primary Progressive Aprosodia can overlap with other related syndromes, especially primary progressive aphasia, frontal lobe dementias and corticobasal degeneration. *No external support was sought or received for this research.*

## ABSTRACT-30

**The Frontal Assessment Battery (Fab) in healthy elderly: influence of education on performance**

Rogério Gomes Beato, Paulo Caramelli

Behavioral and Cognitive Neurology Unit, Faculty of Medicine, Federal University of Minas Gerais, Belo Horizonte, Brazil.

The Frontal Assessment Battery (FAB) has been recently proposed as a diagnostic tool for the identification of patients with frontal lobe syndrome.

### Objectives:

To present the Brazilian version of the FAB and to show preliminary data on the performance of healthy elderly in the battery, correlating with age, education, and scores on the Mini-Mental State Examination (MMSE).

### Methods:

To date, 55 cognitively normal elderly individuals (35 female and 20 male), aged 60-91 years (mean±SD=70.0±6.4) and with educational level ranging from 1-20 years (mean±SD= 8.5±5.7) have been evaluated. The subjects were initially submitted to the MMSE and the Cornell depression scale by one examiner. Subsequently, a second examiner, blind to the subjects’ diagnosis, administered the FAB, where scores were determined for each item and for the total scale. In addition, the subjects were submitted to the following brief cognitive tests: digit span (forward and backward), category fluency (animals/min.), clock drawing and memory test (immediate and delayed recall of ten simple line drawings). All individuals had to perform above education-adjusted cut-off scores in the MMSE, namely, 20 for 1-3 years of schooling, 23 for 4-7 years and 25 for those with 8 or more years of educational level, together with ≤7 points on the Cornell depression scale, in order to exclude depression. Correlations were calculated between FAB total scores and age, gender, educational level and MMSE scores, as well as correlation between FAB items and education. Performance in the other cognitive tests were also tested for correlation with FAB total scores.

### Results:

The mean score±SD on the FAB was 13.0±2.3 (ranging from 7 to 18). There was a significant correlation between total FAB scores and education (r=0.413; p=0.0017) and between total FAB scores and MMSE scores (r=0.43; p=0.001). No correlation was found between total FAB scores and age or gender. Considering the six FAB items separately, two of these (*similarities and conflicting instructions*) independently presented significant correlation with educational level (r=0.37, p=0.005 and r=0.35, p=0.009; respectively). Significant correlations for FAB total scores were also observed for the tests digit span forward (r=0.41; p=0.0018) and backward (r=0.54; p<0.0001), category fluency (r=0.32; p=0.017) and clock drawing (r=0.41; p=0.0019), but not for the memory test.

### Conclusions:

In this group of elderly subjects without evidence of cognitive impairment and depression, the FAB proved to be significantly influenced by education, but not by age or gender. Performance on the FAB was correlated with global cognitive functioning, as measured by the MMSE, and with other frontal lobe tasks (digit span, category fluency and clock drawing) as expected. The completion of this study using a larger sample of controls and inclusion of patients with dementia, will allow the diagnostic accuracy of the FAB, together with cut-off scores for educational level, to be ascertained in our milieu.

## ABSTRACT-31

**Corticobasal degeneration in a behavioral and cognitive neurology outpatient unit: case series of 22 patients**

Renata Areza-Fegyveres, Frota N, Antonio Eduardo Damin, Sonia Maria Dozzi Brucki, Sellitto Claudia Porto, Magaldi R, Santos L, Ono CR , Paulo Caramelli , Carlos Alberto Buchpiguel, Ricardo Nitrini

Behavioral and Cognitive Neurology Unit, Department of Neurology, University of São Paulo School of Medicine; Cognitive Disturbances Reference Center, Hospital das Clínicas of the University of São Paulo School of Medicine; Department of Radiology, University of São Paulo School of Medicine.

Corticobasal degeneration (CBD) can present with marked cognitive impairment. The pattern of deficits can be very variable, but some findings have an important diagnostic value.

### Objective:

To describe the main clinical and cognitive findings from a group of patients with CBD.

### Methods:

Twenty-two patients with clinical diagnosis of CBD who were regularly seen at our outpatient unit were examined and followed for a variable period of time. The group comprised 8 women and 14 men, with ages ranging from 53 to 73 years (median=68 years), educational level ranging from 1 to 16 years (median=6 years) and disease onset between 1 and 4 years from first consultation (median=2 years).

### Results:

Initial Mini-Mental State scores ranged from 17 to 27 points (median=21). Eight patients scored below the normative cut-offs for their respective schooling. Nine patients failed drawing, while eight had temporal and/or spatial disorientation. All patients failed word recall. Category fluency (animals) scores were all below education-adjusted cut-off scores. All patients presented asymmetric parkinsonism and predominant asymmetric apraxic syndrome. Hemineglect was present in 2 patients whereas eight patients showed alien hand phenomena and four, focal limb distonia.

### Conclusion:

Throughout this descriptive study, we have emphasized that CBD can present with marked cognitive and behavioral impairment and that this knowledge is useful to the neurologist.

## ABSTRACT-32

**Apraxia profile in frontotemporal lobar degeneration**

Renata Areza-Fegyveres , Frota NAF, Cavalcante K, Sonia Maria Dozzi Brucki, Claudia Sellitto Porto, Ricardo Nitrini

Behavioral and Cognitive Neurology Unit, Department of Neurology, University of São Paulo School of Medicine; Cognitive Disturbances Reference Center, Hospital das Clínicas of the University of São Paulo School of Medicine.

In routine daily clinical practice, clinicians often face difficulty reaching diagnosis when involving corticobasal degeneration (CBD), progressive supranuclear palsy, frontotemporal dementia (FTD) and the other frontotemporal lobar degenerations. Praxis deficits seem to be typical of CBD, but occasionally we encounter overlapping clinical syndromes with apraxia in other diseases.

### Objective:

To analyze praxis and behavioral alterations in patients with CBD and FTD.

### Method:

Five patients with CBD, and 5 with FTD, will be selected from the out-patient clinic of the Cognitive and Behavioral Neurology Unit of the Hospital das Clínicas of the University of São Paulo and from the Cognitive Disturbances Reference Center. They will be evaluated by trained examiners with a structured protocol, consisting of identification and demographic data, retrospective data collected from their charts and scales, and tests to measure cognitive performance and behavioral impairments including the Mini-Mental State Exam, Neuropsychiatric Inventory, Pfeffer Daily Activities Scale, Frontal Assessment Battery, Drawing Memory test and Apraxia Battery.

## ABSTRACT-33

**Theory of mind and executive functions in frontotemporal lobar degeneration and Alzheimer disease**

René Martins Viana, Valéria Santoro Bahia, Ricardo Nitrini

Behavioral and Cognitive Neurology Unit, Department of Neurology, University of São Paulo School of Medicine;, Cognitive Disturbances Reference Center, Hospital das Clínicas of the University of São Paulo School of Medicine; Department of Radiology, University of São Paulo School of Medicine.

The term Theory of Mind (ToM) was first cited in 1978. ToM is the ability to attribute mental states – convictions, feelings, thoughts, etc. – to oneself and others. ToM and Executive Functions (EF) are skills needed for proper social functioning. Lesions in frontal lobes may impair the functioning of ToM and EF, occurring in the most frequent subtypes of Frontotemporal Lobar Degeneration (FTLD): frontotemporal dementia, semantic dementia and progressive nonfluent aphasia, as well as Alzheimer disease. The initial symptoms of FTLD and AD are generally divergent: EF seems to be affected earlier in FTLD than in AD, which allows us to investigate if EF and ToM are inter-dependent.

### Objectives:

To assess the performance of FTLD and AD patients in tasks of ToM (verbal and visual) and EF, and to verify if EF interferes in ToM tasks.

### Methods:

10 FTLD, 10 AD patients, and 10 healthy controls. Diagnostic criteria for both groups: the Brief Battery, DAD – Disability Assessment for Dementia, NPI – Neuropsychiatric Inventory, DRS – Mattis Dementia Rating Scale and the Cornell Scale for Depression in Dementia. FTLD patients must meet the research criteria established by Neary, Snowden, Gustafson et al. (1998), and AD patients will be assessed by the DSM-IV and NINCDS/ADRDA criterions. Neuropsychological assessment of EF shall use these tests: Similarities – WAIS-III, WCST, Digit Span (WAIS-III), Trail Making Test, Stroop Test; ToM tasks: Faux Pas, Picture Sequencing Test, 1^st^ and 2^nd^ order false-belief ToM tests. All patients will be assessed individually, following the same test order.

## ABSTRACT-34

**Transient global amnesia and semantic dementia**

Ricardo Nitrini, Mirna Hosogi Senaha, Paulo Caramelli

Behavioral and Cognitive Neurology Unit, Department of Neurology, University of São Paulo School of Medicine;, Cognitive Disturbances Reference Center, Hospital das Clínicas of the University of São Paulo School of Medicine; Department of Radiology, University of São Paulo School of Medicine.

Transient global amnesia (TGA) is usually regarded as a benign condition, but it may be a risk factor for stroke and even for dementia. Two patients with semantic dementia (SD) suffered episodes that were retrospectively diagnosed as TGA.

### Case 1:

A 66 year-old retired teacher presented with a two-year history of progressive word-finding difficulties and impairment of word comprehension that one year later fulfilled the criteria for the diagnosis of SD. Seven years before the beginning of the language disturbances she suffered one episode with the characteristics of TGA, which recurred one year later.

### Case 2:

A 75 year-old businessman presented with a three-year history of fluent progressive aphasia with naming and reading difficulties. In the year before the first consultation he had experienced three episodes with the characteristics of TGA. In both cases, MRI disclosed severe left temporal lobe atrophy, which was more prominent in the anterior part, but also involved the hippocampal formation.

### Discussion:

As far as we know, TGA has not been considered more frequent in patients with SD. Although the pathophysiology of TGA is still disputed, the temporal lobe is probably the main implicated structure, which may link both conditions. We tentatively raise three hypotheses for our findings: a fortuitous association; TGA may predispose to semantic dementia; the presence of left temporal lobe pathology may render the patient more susceptible to the disturbance that causes TGA.

### Conclusion:

Further investigations should look for the occurrence of this association and, if it is present, for a causal relationship.

## ABSTRACT-35

**Temporal lobe epilepsy and primary progressive aphasia**

Ricardo Nitrini^1^, Mirna Hosogi Senaha^1^, Quagliato E^2^, Cláudia Sellitto Porto^1^

^1^Behavioral and Cognitive Neurology Unit, Department of Neurology, University of São Paulo School of Medicine;, Cognitive Disturbances Reference Center, Hospital das Clínicas of the University of São Paulo School of Medicine; Department of Radiology, University of São Paulo School of Medicine.

^2^Department of Neurology, University of Campinas (UNICAMP), Brazil.

The causes of the higher vulnerability of the left cerebral hemisphere, particularly of the left temporal lobe, in the pathogenesis of primary progressive aphasia (PPA) are still unknown. Developmental dyslexia and childhood injury to the left temporal lobe have been described as conditions that may predispose to PPA. As far as we know, temporal lobe epilepsy has not been included among them.

### Case Report:

A 60 year-old man, a retired engineer, manifested generalized tonic-clonic seizures since he was 4 years-old. In the adulthood, complex partial seizures became more frequent, characterized by epigastric aura, déjà-vu sensation and gestural automatisms. Electroencephalograms showed epileptic activity in the left temporal lobe. Since he was 41 years-old, he has been treated with carbamazepine, with occasional seizures. At the age of 56, he started to have relentless progressive speech problems, with word finding and verbal comprehension difficulties. In spite of these, he continued to drive his car, to play tennis with friends, and to take care of his own medications. Four years after the beginning of the language problems, he scored 23 in the MMSE, his performance was in the 25^th^ percentile in the Raven Progressive Matrices, verbal comprehension and expression were impaired, but recent autobiographic and semantic memories were apparently preserved. MRI showed slight enlargement of the sulci, and lower volume and sclerosis of the left hippocampus. SPECT disclosed hypoperfusion in the left cerebral hemisphere.

### Conclusion:

This association of left mesial temporal sclerosis and PPA may be casual or have causal relationship.

